# Chemical-imaging-guided optical manipulation of biomolecules

**DOI:** 10.3389/fchem.2023.1198670

**Published:** 2023-05-05

**Authors:** Matthew G. Clark, Seohee Ma, Shivam Mahapatra, Karsten J. Mohn, Chi Zhang

**Affiliations:** ^1^ Department of Chemistry, West Lafayette, IN, United States; ^2^ Purdue Center for Cancer Research, West Lafayette, IN, United States; ^3^ Purdue Institute of Inflammation, Immunology and Infectious Disease, Purdue University, West Lafayette, IN, United States

**Keywords:** chemical imaging, optical control, laser-scanning microscopy, biological manipulation, fluorescence microscopy

## Abstract

Chemical imaging via advanced optical microscopy technologies has revealed remarkable details of biomolecules in living specimens. However, the ways to control chemical processes in biological samples remain preliminary. The lack of appropriate methods to spatially regulate chemical reactions in live cells in real-time prevents investigation of site-specific molecular behaviors and biological functions. Chemical- and site-specific control of biomolecules requires the detection of chemicals with high specificity and spatially precise modulation of chemical reactions. Laser-scanning optical microscopes offer great platforms for high-speed chemical detection. A closed-loop feedback control system, when paired with a laser scanning microscope, allows real-time precision opto-control (RPOC) of chemical processes for dynamic molecular targets in live cells. In this perspective, we briefly review recent advancements in chemical imaging based on laser scanning microscopy, summarize methods developed for precise optical manipulation, and highlight a recently developed RPOC technology. Furthermore, we discuss future directions of precision opto-control of biomolecules.

## Introduction

Over the past few decades, the field of microscopy has experienced rapid growth with improved resolution, better contrast, higher sensitivity, and faster speed. Revealing the morphological features of the specimen can no longer quench the persistent thirst for a more wholesome understanding of biological functionality. Visualizing the chemical compositions of the sample becomes a necessity for fundamental biological science. Chemical signatures on the microscopic scale also enable new possibilities such as the diagnosis of pathological transitions, the designing of better therapies, and the discovery of new paths of drug resistance. Among many chemical imaging methods, optical microscopy, being the most widely used chemical mapping tool for biological studies, has unique advantages in chemical specificity and spatial resolution. Typically, chemical contrasts in optical microscopy arise from fluorescence labeling, light absorption, and Raman scattering. Technology breakthroughs in all these fields open tremendous new opportunities and directions in biological sciences.

Despite such advancements in microscopy, a microscope does not offer more than passive observations of a biological specimen. To better understand bio-functions and mechanisms, it is critical to actively partake in the chemical processes of samples and manipulate the behaviors of biomolecules. Such a capability, however, is very rudimentary at the current stage. The conventional way of incubating cells with exogenous chemicals does not allow for control of the spatial diffusion of chemicals. The introduced compound can impact multiple pathways and targets, giving convoluted results. Simple modifications of advanced microscopy platforms allow for precise interrogation of spatially defined structures with lasers; however, this usually requires *a priori* knowledge of the sample and image analysis. The lack of technology to spatially and selectively control chemical processes has limited the understanding of spatial functions of molecules in biological processes. Recent developments of laser-based opto-control technologies hold promise to revolutionize biological science by controlling biomolecular behaviors precisely, selectively, and in real time.

In this perspective article, we first briefly discuss recent advancements in optical imaging technologies for the generation of chemical contrasts. Then, we give an overview of laser-based technologies for optical control and manipulation of biomolecules. More emphasis is given to a recently developed real-time precision opto-control (RPOC) technology. Finally, the future of opto-control of biomolecular behaviors is projected.

## Optical imaging technologies for chemical analysis

**FIGURE 1:**
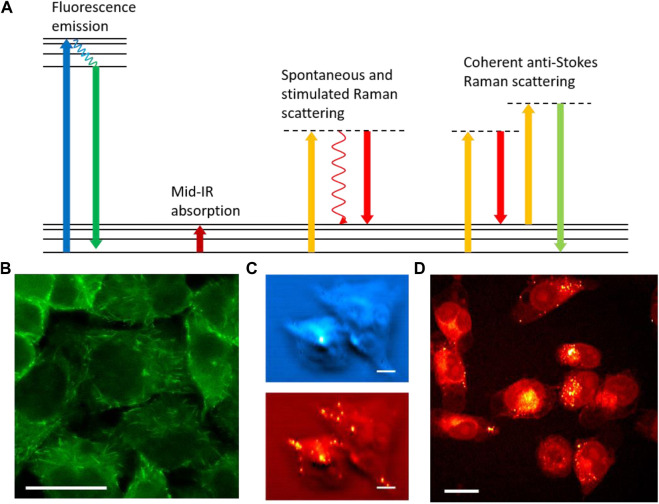
Optical imaging for chemical analysis of biological samples. **(A)** Energy diagrams of fluorescence emission, mid-IR absorption, spontaneous and stimulated Raman scattering, and coherent anti-Stokes Raman scattering processes. **(B)** Fluorescence imaging of EB3 protein in EB3-EGFP transfected HeLa cells. **(C)** Photothermal IR imaging of Atglistatin (blue) and lipid droplets (red) in MIA PaCa2 cells. Scale bars are 20 µm. **(D)** Stimulated Raman scattering imaging of lipids in MIA PaCa2 cells. Panel C adapted with permission from reference (Zhang et al., 2016).

To perform chemical-specific optical manipulation, it is critical to detect the chemicals of interest in biological samples with high selectivity. The most widely applied optical microscopy modalities to map chemical compositions in live cells include fluorescence, infrared absorption, and Raman scattering (Figure 1A).

Fluorescence microscopy was developed in the early 20th century. Fluorescence rapidly opened up possibilities for labeling specific targets and deriving chemical information through the labeling. Two crucial breakthroughs were the development of fluorescent antibody labeling (Coons et al., 1941; Coons et al., 1942) and fluorescent protein cloning (Prasher et al., 1992; Chalfie et al., 1994), both of which offer unparalleled specificity for proteins. The specificity of the former originates from the affinity of the conjugated antibody to its antigen, while the latter stems from targeted gene editing and protein expression. With various fluorescence protein variants and gene editing techniques being developed, genetic transfection makes visualizing various proteins in live processes a reality. Figure 1B shows a fluorescence image of end-binding protein 3 (EB3) transfected with enhanced green fluorescence protein (EGFP) in HeLa cells. Real-time monitoring of EB3 that binds to the plus end of the microtubule is a powerful method to study tubulin polymerization and cytoskeleton dynamics in live cells. Over the years, advanced modalities such as confocal (Marvin, 1961), light sheet (Huisken et al., 2004), total internal reflection fluorescence (TIRF) (Axelrod, 1981; Axelrod, 2001), fluorescence lifetime imaging microscopy (FLIM) (Bastiaens and Squire, 1999), Förster resonance energy transfer (FRET) (Clegg, 1995), and fluorescence *in situ* hybridization (FISH) (Langer-Safer et al., 1982) have been added with fluorescence microscopy to deliver more information in chemical composition and molecular dynamics. These modalities served as excellent tools for understanding fundamental biochemistry and visualizing disease pathogenesis. The high quantum yield of fluorophores and low background enable single molecule detection using fluorescence microscopy. This unmatchable sensitivity allows for resolving single-molecule activities and dynamics (Shashkova and Leake, 2017). Furthermore, super-resolution fluorescence microscopy technologies such as stimulated emission depletion (STED) (Hell and Wichmann, 1994), photoactivatable localization microscopy (PALM) (Betzig et al., 2006; Hess et al., 2006), stochastic optical reconstruction microscopy (STORM) (Rust et al., 2006), and structured illumination microscopy (SIM) (Pullman et al., 2016) break the optical diffraction limit to reveal fine structures with spatial resolution on the nanometer scale. The development of multiphoton excitation fluorescence further extends fluorescence microscopy for deep tissue imaging (Denk et al., 1990; So et al., 2000). Fluorescence microscopy is the most widely applied optical technology in biological science for chemical imaging.

Unlike fluorescence, in which chemical specificity majorly resides in the labeling mechanism, the chemical information from infrared (IR) absorption can be readily extracted by absorption transitions of chemical bonds. IR transitions are allowed when a change of dipole is induced during vibration. As a label-free imaging method, IR provides rich information on chemical compositions, especially for small molecules and metabolites (Katon, 1996; Bellisola and Sorio, 2012). To avoid strong absorption from water and CO_2_, the mid-IR beam path is usually purged with nitrogen gas. Still, the strong water absorption limits the penetration depth of mid-IR beams to reach deeper layers of biological samples. Due to the use of mid-IR sources which have much longer wavelengths compared to visible light, the spatial resolution of conventional IR microscopy is much lower than fluorescence microscopy. To improve spatial resolution, several advanced superresolution far-field IR imaging technologies have been developed in recent years. Photothermal IR microscopy is among the most recently developed IR imaging methods (Zhang et al., 2016; Xia et al., 2022). The mid-IR absorption in photothermal IR microscopy creates a thermal lens that alters the local refractive indices of the sample. Such a thermal lens can change the deflection or optical path length of a visible laser beam that is used to probe such changes (Zhang et al., 2016; Zhang et al., 2019). The spatial resolution is defined by the diffraction limit of the visible beam which is smaller than the IR diffraction limit. Figure 1C shows photothermal IR images of Atglistatin and lipid compositions in MIA PaCa2 cells with sub-micron resolution. In addition, the differences in thermal relaxation at the center and edge of the thermal lens allow for a resolution beyond the diffraction limit of the visible laser (Fu et al., 2023). Aside from IR absorption, transient absorption microscopy, which probes the lifetime of electronically excited states, also gives rich chemical information in the time domain to differentiate strong light absorbers (Fu et al., 2008; Zhu and Cheng, 2020).

Besides fluorescence and IR microscopy, Raman scattering is also widely applied for chemical imaging. Raman scattering occurs when an incident photon is inelastically scattered upon interacting with a sample, and the change in photon energy is equivalent to that of a vibrational transition (Petry et al., 2003). The use of visible laser for Raman spectroscopy provides good spatial resolution and largely avoids strong water absorption as in IR spectroscopy. Therefore, Raman analysis can detect analytes much deeper under the surface compared to mid-IR measurement. However, spontaneous Raman scattering is a much weaker process (typically 10^−12^ times weaker) compared to absorption and fluorescence. Consequently, a long signal integration time is needed for spontaneous Raman spectroscopy. Such as acquisition time is fast enough for many applications in spectral analysis but insufficient in biological imaging of dynamic samples. To improve signal levels and imaging speed, advanced Raman techniques have been developed. Resonance enhancement can improve Raman signal levels by using laser wavelengths close to the electronic resonance, albeit at the price of potential sample damage and stronger fluorescence background (Strommen and Nakamoto, 1977). Surface-enhanced Raman spectroscopy (SERS) provides strong Raman signal amplification but requires hot spots created by metal surfaces (Stiles et al., 2008). Tip-enhanced Raman spectroscopy (TERS) is a near-field technology that gives label-free vibrational analyses of samples at nanometer resolution (Stöckle et al., 2000). Recently developed coherent Raman scattering methods, particularly coherent anti-Stokes Raman scattering (CARS) and stimulated Raman scattering (SRS) processes, have demonstrated far-field high-speed Raman imaging of biological samples (Cheng and Xie, 2004; Freudiger et al., 2008; Ozeki et al., 2009; Zhang et al., 2015; Yue and Cheng, 2016; Prince et al., 2017). Coherent Raman processes give million-fold Raman signal enhancement, making Raman imaging as fast as fluorescence microscopy. In Raman imaging, chemical contrasts are generated from chemical bonds. This makes Raman a label-free technique, which, similar to IR, yields it especially powerful for analyzing small molecules such as metabolites and pharmaceutical compounds. Figure 1D shows an image of lipids in MIA PaCa2 cells revealed by SRS microscopy. However, direct analysis of chemical bonds does not give enough information to identify large molecules such as proteins. Despite the development of advanced Raman tags to label specific proteins, similar to those used in fluorescence (Wei et al., 2014), the Raman sensitivity still falls behind fluorescence by several orders of magnitude. Comparing Raman tag labeling with fluorescence labeling, the former gives better multiplexing due to the narrow Raman peaks (Wei et al., 2017) and also less alteration of molecular functionality due to the small size of the tags (Palonpon et al., 2013), while the latter offers much better sensitivity and better selectivity for proteins. On the instrumentation side, fluorescence microscopes are more mature and cost-effective compared to coherent Raman microscopes.

## Optical technologies for controlling chemical processes

Compared to conventional chemical control methods, optical control renders fast response and high spatial precision. Laser-based technologies offer analysts unique and powerful approaches to probe and manipulate chemical interactions in biological systems at sub-micron spatial resolution scales with minimal perturbation to the system. Although the design of these technologies may differ, one key similarity is the ease of integration with imaging systems. Coupling these systems allows for precise chemical control and remarkable temporal precision.

**FIGURE 2:**
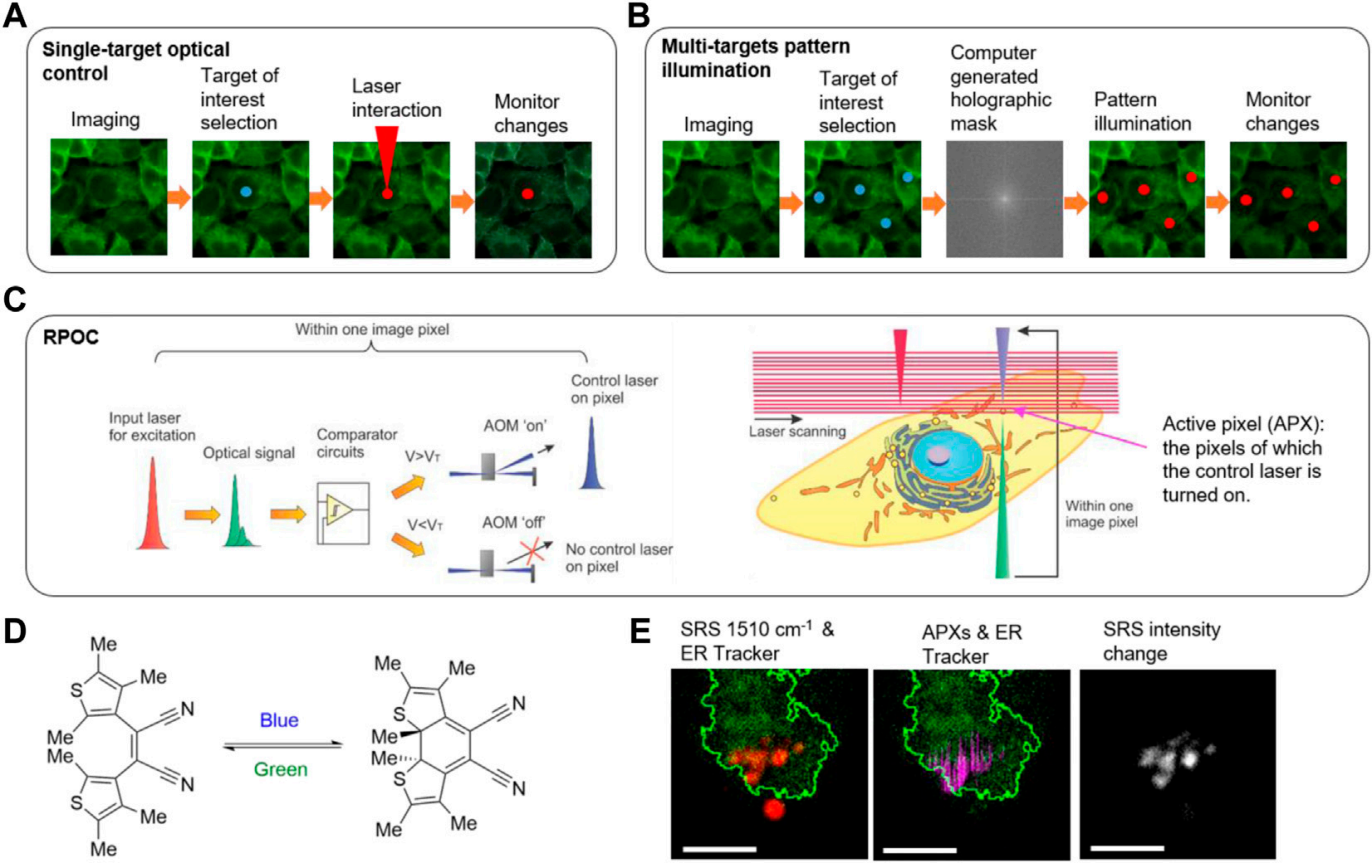
Optical control technologies. **(A)** The workflow of imaging-guided single-spot optical manipulation. **(B)** The workflow of imaging-guided multi-spot optical control based on pattern illumination. **(C)** The principle of real-time precision opto-control (RPOC). AOM: acousto-optic modulator; V_T_: threshold voltage. **(D)** A photochromic molecule *cis*−1,2-dicyano1,2-bis(2,4,5-trimethyl-3-thienyl)ethane (CMTE) and the isomeric changes induced by optical exposure at different wavelengths. **(E)** RPOC converts CMTE in the endoplasmic reticulum (ER) from the closed form to the open form while not affecting the CMTE outside the boundary of ER. Red: SRS signals from CMTE; Green: ER Tracker two-photon excitation fluorescence signals; Magenta: active pixels; Greyscale: SRS signal change after RPOC. Scale bars are 5 µm. Panels **(C–E)** adapted with permission from reference (Clark et al., 2022).

One of the most popular applications of laser manipulation technology in biology and medicine is the ablation of tissue. Continuous-wave (CW) lasers have been employed to carry out ablation for biological samples since 1963 (Rosan et al., 1963). The thermal effect created by CW laser ablation reduces the spatial precision and induces photodamage of unwanted targets. Ultrashort laser pulses enable precise and minimally invasive ablation for the microsurgery of sub-micron-scale features in living organisms (Vogel et al., 2005; Steinmeyer et al., 2010). They have been utilized to investigate the function of mitochondria (Watanabe et al., 2004), excise microtubules (Botvinick et al., 2004), manipulate DNA transfection (Tsukakoshi et al., 1984), control ER calcium flux (He et al., 2012), and induce cell senescence (Zhao et al., 2022), only to name a few. Imaging and analysis are prerequisites to selecting the targets of interest and guiding the ablation laser for optical manipulation (Figure 2A).

Besides photoablation, optical light can also activate photosensitive molecules. Photoactivation describes the use of light to either irreversibly or reversibly control a desired chemical reaction. Of these processes, two major techniques are typically employed: site-specific photo-uncaging for the release of active small molecules and molecular photoswitching for a controllable manipulation of chemical reactions. Photoremovable or photolabile chemical groups are the key aspect of molecular photo-uncaging (Pelliccioli and Wirz, 2002; Klán et al., 2013). Through spatiotemporally gated light illumination, protecting group-functionalized inactive molecular species can be “uncaged” and activated for precise control over the localization of chemical reactions in dynamic biological samples. The types of protecting groups commonly used are vast; however, there are universal criteria that must be considered for their application. These include strong absorption at >360 nm to minimize cellular damage, a high quantum yield for efficient photo-release, high solubility or permeation in the target media, and minimally reactive photochemical byproducts with transparency at the excitation wavelength. Two-photon-based excitation schemes have grown more popular for photo-uncaging as the wavelength choice decreases phototoxicity, prevents out-of-plane excitation events, and improves the penetration depth of the excitation laser (Denk et al., 1994; Gagey et al., 2007).

Photoswitching describes the method by which absorption of electromagnetic radiation can reversibly interconvert a target chemical species between different functional isomers (Alfimov et al., 2003; Gorostiza et al., 2007; Harris et al., 2018). This allows the molecule to be turned “on” and “off” selectively at will via controlled illumination between stable and metastable isomers. Mechanistically, this is comprised of two major types of isomerization events: *cis* to *trans* conversions, or ring opening-closing mechanism upon irradiation (Alfimov et al., 2003; Gorostiza et al., 2007). Typically, one wavelength of light is used to convert the molecular photoswitch into a metastable isomer form, which can then be converted back either using another wavelength of light or via kinetic means, depending on the thermal stability of its metastable state. Similar to photo-uncaging, two-photon absorption has also been utilized to manipulate photoswitches (Carling et al., 2009; Dong et al., 2015; Muñoz-Rugeles et al., 2020). The advantage of the switching process lies in the potential to selectively steer and reverse chemical reactions in biological samples. Photoswitchable proteins such as Dronpa (Habuchi et al., 2005; Flors et al., 2007; Zhou et al., 2012) and cyanine dyes such as Cy3-Cy5 (Bates et al., 2005; Rust et al., 2006) have been widely used in superresolution localization microscopy.

The aforementioned methodologies use exogenous molecular species for cellular manipulation. Optogenetics, in contrast, combines the genetic expression of photoswitches in protein scaffold for light control (Deisseroth, 2011; Yizhar et al., 2011). Initial research interest was focused on the expression and applications of microbial opsins, though, recently, a myriad of non-opsin-based photoreceptors have been developed for more tunable optical properties (Deisseroth, 2015; Ma et al., 2017; Chia et al., 2022). This technology has found its place in neurobiology, primarily used as a tool to understand and map out neuronal activities. Due to the scattering nature of thick brain tissue, multiple light simulation tools have been developed to address penetration considerations, including implantable fiber optics and waveguides (Pisanello et al., 2014). Patterned illumination strategies have also been developed using spatial light modulators (SLM) (i.e., liquid crystal or microelectromechanical) or digital micromirror devices (DMD) for parallel stimulation of multiple spatially distributed targets (Nikolenko et al., 2008; Arrenberg et al., 2010). Liquid crystal SLMs manipulate the phase of the light using computer-generated holography (CGH) masks for pattern illumination (Papagiakoumou, 2013; Pégard et al., 2017). DMDs spatially modulate the light amplitude instead but achieve similar performance at a slight cost of lateral resolution. Both methods produce light patterns by spatial interference. These pattern illumination methods are also applied to other fields such as 3D microprinting based on photoactivatable polymerization (Chu et al., 2018; Somers et al., 2021).

Despite the development of advanced structure illumination technologies based on SLM and DMDs, *a priori* knowledge of the sample is required to generate the CGH masks for producing light patterns (Figure 2B). Such a requirement slows down the response time and therefore makes these methods not capable of following intracellular molecules with high dynamics. Furthermore, interference-based optical pattern generation is not clean, with laser speckles presenting in undesired locations, especially for nonperiodic and random patterns. Recently, the Zhang group demonstrated a real-time precision opto-control (RPOC) technology that is capable of controlling molecular targets at sub-micron precision in real-time for highly dynamic living samples (Clark et al., 2022). RPOC is based on a laser scanning microscope. During laser scanning, optical signals carrying chemical information from the sample are generated and detected at pixels of interest. The desired signals, after real-time processing, command an acousto-optic modulator (AOM) to rapidly couple another laser beam to trigger chemical reactions at the same pixel. Chemical information from RPOC can be generated from fluorescence, SRS, and absorption processes. RPOC has a sub-microsecond response time, high chemical specificity, and sub-micron spatial precision. Figure 2C shows the principle of RPOC. An active pixel (APX) in RPOC is defined as the pixel on which the control laser is turned on. A real-time closed-loop feedback comparator system is used for determining APXs associated with chemical contrasts. APXs can be selected for any random pattern formed by chemical distributions in the specimen, with higher-order digital logic functions available. Figure 2D shows the structures of a photochromic molecule *cis*−1,2-dicyano1,2-bis(2,4,5-trimethyl-3-thienyl)ethene (CMTE), which can be controlled by lasers. RPOC allows changing the chemical structures of CMTE in the ER while not affecting the molecules outside of ER despite being spatially close (Figure 2E). Furthermore, used with a photoswitchable tubulin polymerization inhibitor, RPOC was applied to control the subcellular tubulin polymerization and lipid droplet active transport by targeting site-specific microtubule networks (Clark et al., 2022). RPOC technology allows biologists to monitor chemical targets of interest using advanced chemical imaging modalities and simultaneously control only the activities of biomolecules associated with these targets using lasers and photoswitchable compounds. This capability can render new approaches to interrogate site-specific chemical reactions and biomolecular functions. Opportunities and challenges impend in this nascent field bridging optical engineering, chemistry, and biological science.

## Discussion

Advanced optical technologies enable flexible manipulation of lasers with biological samples to control chemical reactions. Fast laser scanning and a real-time feedback system allow for the precise determination of APXs at targets of interest without affecting unwanted locations. The APXs can be selected for any randomly distributed chemicals if sufficient signal-to-noise ratios can be achieved. The key advantages of this approach compared to the pattern illumination methods based on SLM or DMD are the instantaneous response time and clean APXs for any random pattern. RPOC allows to trace and control chemical reactions of highly dynamic molecules of interest in living samples. The RPOC prototype is based on a 2D galvanometer scanner with a fast axis speed of 1 kHz. The speed of RPOC can be further improved by using faster scanners such as a resonant mirror or a polygonal mirror for molecules with a faster moving speed. The current RPOC system only commands a single control laser wavelength using an AOM. Applying multiple AOMs to separate lasers or an acousto-optic tunable filter (AOTF) after combining all wavelengths would simultaneously manage multiple laser wavelengths. Such a capability allows active regulation and selection of the state of photoswitchable compounds at different pixels or the simultaneous control of multiple photoswitchable molecules.

RPOC can be integrated with any laser scanning microscope. The use of a high-power femtosecond laser, despite enabling multiphoton excitation fluorescence and coherent Raman scattering modalities, limits the application of RPOC in biological science due to the high laser cost. A CW laser-based RPOC technology would greatly reduce the system complexity and cost, and can be integrated with laser scanning confocal microscopes for precision opto-control. In this case, fluorescence signals will be majorly used for extracting chemical information from the sample to guide the control lasers.

The opto-control laser beam can be flexible for different processes. Advanced photoswitchable or photoactivatable compounds can be developed to pair with RPOC for precision control of chemical processes in live cells at sub-micron precision and in real-time. RPOC can also scale up spatially to control chemical processes in larger fields of view for precise photodynamic therapy or treatment.

With continuous advancements in optical engineering and laser technology, we expect that precision opto-control will enable new methods for steering chemical reactions and controlling biomolecular behaviors in a site-specific manner. This capability will lead to new insights into biological functions and drug-target interactions for biomedical sciences. Actively determining the fate of a biological sample will become a more popular and powerful approach for life scientists to decipher the mystery of life.

## Data Availability

The original contributions presented in the study are included in the article/Supplementary Material, further inquiries can be directed to the corresponding author.
