# Supplementary material for: Chemical-imaging-guided optical manipulation of biomolecules
Source: Front Chem. 2023 May 5;11:1198670. doi: 10.3389/fchem.2023.1198670 (PMC10196011; doi:10.3389/fchem.2023.1198670)
Supplement: Supplementary file 1 [file DataSheet1.docx]

Supplementary materials

Chemical-imaging-guided optical manipulation of biomolecules

Matthew G. Clark^1^, Seohee Ma^1^, Shivam Mahapatra^1^, Karsten J. Mohn^1^ and Chi Zhang^1,2,3*^

^1^Department of Chemistry, West Lafayette, IN, United States, ^2^Purdue Center for Cancer Research, West Lafayette, IN, United States, ^3^Purdue Institute of Inflammation, Immunology and Infectious Disease, Purdue University, West Lafayette, IN, United States

Supplementary contents

Figure 1B is acquired using a laser-scanning confocal fluorescence microscope (LSM 510, Zeiss). HeLa cells expressing EB3-EGFP are purchased from BioHippo and cultured in DMEM medium with 10% Fetal Bovine Serum and 1% Penicillin-Streptomycin. The laser wavelength for excitation is 488 nm at 5 mW. The signal acquisition optical filter is 505 nm long-pass. The pixel dwell time is 2 microseconds.

Figure 1D is acquired using a lab-built stimulated Raman scattering (SRS) microscope. The pump laser pulse has a center frequency of 800 nm, pulse width of 3.4 ps, and average power of 15 mW. The Stoke pulse has a center frequency of 1045 nm, pulse width of 1.8 ps, and average power of 30 mW. The pixel dwell time is 10 microseconds. The laser has a repetition rate of 80 MHz. More details of the SRS instrument can be found in the reference:

Clark, M. G., Gonzalez, G. A., Luo, Y., Aldana-Mendoza, J. A., Carlsen, M. S., Eakins, G., et al. (2022). Real-time precision opto-control of chemical processes in live cells. *Nat. Commun.* 13, 4343. doi:10.1038/s41467-022-32071-z
